# Personalized physiotherapy effect modeling with continuous causal machine learning

**DOI:** 10.3389/fdgth.2026.1793661

**Published:** 2026-10-27

**Authors:** Louk van Remmerden, Eline Kooijmans, Shujian Yu, Karlijn Joling, Emiel Hoogendijk, Caitlin McArthur, Mark Hoogendoorn, Hein van Hout

**Affiliations:** 1 Vrije Universiteit Amsterdam, Department of Computer Science, Amsterdam, Netherlands; 2 Amsterdam UMC Location Vrije Universiteit Amsterdam, Department of General Practice, Amsterdam, Netherlands; 3 Amsterdam Public Health, Aging & Later Life, Amsterdam, Netherlands; 4 School of Physiotherapy, Faculty of Health, Dalhousie University, Halifax, NS, Canada

**Keywords:** causal inference, continuous treatment estimator, dose–response modeling, non-pharmacological intervention, personalized medicine, physiotherapy

## Abstract

**Introduction:**

Estimating how physiotherapy (PT) intensity influences functional independence is central to personalized care planning for older adults. However, most existing causal inference approaches treat PT as a binary intervention, which requires collapsing a naturally continuous measure of treatment intensity and limits the ability to characterize nuanced dose–response relationships.

**Methods:**

To address this limitation, we use continuous causal inference to estimate individualized continuous dose–response curves for PT. This approach enables granular assessment of treatment effects across therapy intensities and supports the identification of optimal treatment doses. The model is trained on Canadian Community Care data and externally validated using an independent Canadian Home Care cohort.

**Results:**

Across factual, proxy counterfactual, and external validation settings, our approach generally achieved lower prediction error on activities of daily living outcomes, as measured by root mean squared error (RMSE), than standard baselines. In particular, we attained factual and proxy counterfactual RMSEs of 3.79 and 3.89, respectively, compared with 4.09 and 6.49 for linear regression, while also demonstrating strong external generalization.

**Conclusion:**

Continuous causal modeling of individualized dose–response relationships provides a principled approach to estimating physiotherapy intensity effects on functional independence, with direct implications for personalized rehabilitation planning in older adults.

## 1 Introduction

Non-pharmacological interventions (NPIs) are increasingly recognized as essential components of modern healthcare, offering less invasive and often more accessible alternatives to drug-based treatments (1). They have been evaluated across a wide range of clinical contexts, including depression (2), cognitive behavioral therapy for insomnia (3), and physical therapies during pregnancy (4). In older adults, NPIs have been studied for preventing falls (5) and managing incontinence (6). As NPIs continue to play a larger role in routine care, developing reliable methods to understand and predict their effects has become more important.

Due to its effectiveness, physiotherapy (PT) is a widely used non-pharmacological intervention that aims to improve, maintain, and restore individuals’ ability to move and function optimally.^1^ Its effectiveness is largely attributed to its role in maintaining muscle function and cardiorespiratory fitness, thereby supporting functional ability and the management of chronic disease (7, 8). PT is delivered across the lifespan and is used when movement is affected by aging, injury, illness, disability, or environmental factors. The overarching goal is to promote long-term functional improvement, prevent future injury, and address underlying impairments rather than merely alleviating symptoms. Nevertheless, PT prescription remains challenging because its effects differ considerably across individuals (9). This response variation has sparked interest among researchers to develop decision-support models which can aid clinicians in predicting the effect of PT on an individual level (1, 10). Addressing this challenge requires methods that can explicitly account for both treatment effect heterogeneity and confounding present in observational healthcare data.

Causal machine learning methods in particular have shown promise in modeling individual treatment effects by providing a principled framework for effect estimation through confounder adjustment (11). Such methods have already been applied successfully in settings aimed at predicting the effect of PT on functional independence (12), as well as more broadly to the estimation of *pharmacological* treatment effects in older adults (13). Despite these advances, most existing applications rely on simplified, binary representations of treatment, using causal ML approaches such as meta-learners (14) or counterfactual regression (15).

However, binary treatment formulations provide a limited representation of physiotherapy practice. To model the effect of physiotherapy on functional independence in a more realistic manner, we require a less constrained, flexible treatment modeling approach. After all, in practice, physiotherapy is rarely administered in standardized doses; instead, clinicians routinely tailor the duration, intensity, and frequency of sessions to individual patient needs (1, 2, 16, 17). Moreover, as noted by van Remmerden et al. (12), binary causal treatment formulations require discretization of continuous treatment variables, for which the choice of cut-off is often unclear and can introduce bias into treatment effect estimation. Together, these limitations motivate the need for causal frameworks that can directly accommodate continuously valued treatments without imposing *ad hoc* thresholds.

Recent work in the machine learning community has begun to address this gap (18, 19) by introducing causal algorithms capable of estimating treatment effects over a continuous treatment range. These approaches enable the estimation of continuous dose–response functions, which map each feasible treatment intensity to its expected outcome, as illustrated in Figure 1. Learning individualized dose–response curves, in turn, allows clinicians to identify an *optimal* treatment intensity, with potential gains in clinical efficiency and cost-effectiveness. Building on these advances, we study the use of continuous-treatment causal models in the context of physiotherapy, a real-world clinical setting in which treatment intensity is inherently continuous and individualized.

**Figure 1:**
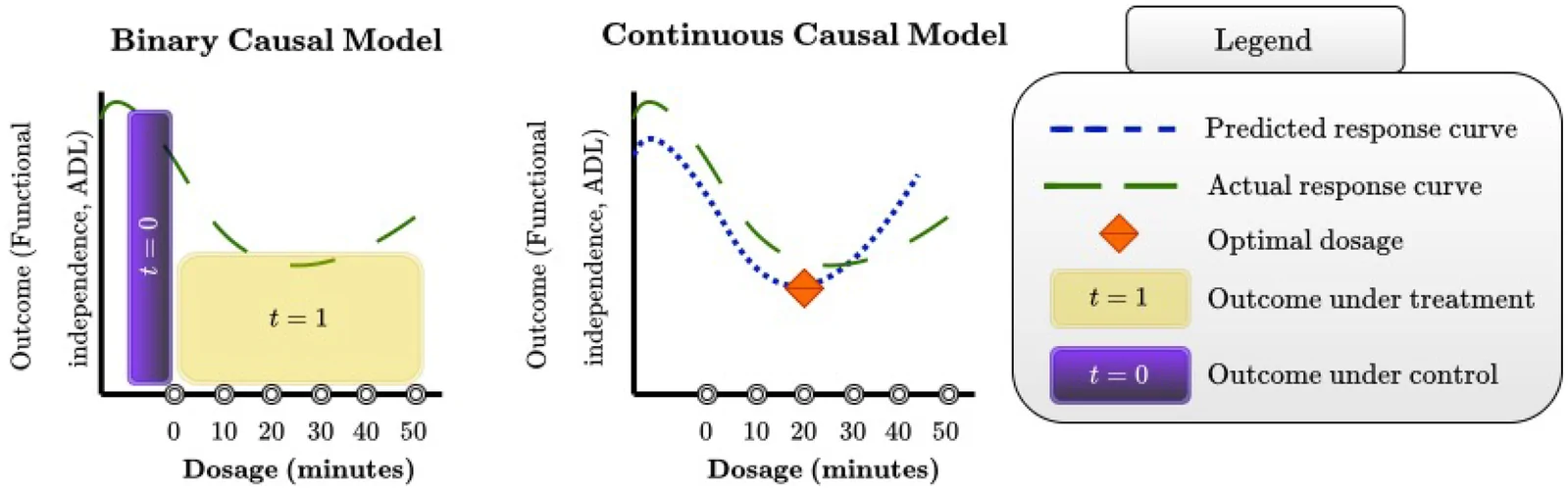
Illustrative example of the two causal modeling paradigms: binary and continuous. t denotes the (binary) treatment, with 1 indicating that treatment was prescribed and 0 indicating that it was not. Left: the BinaryCausalModel setting, where for a given individual only two predictions can be recovered: (1) the outcome when treatment is 0 (shown in purple, where no minutes of physiotherapy have been prescribed), and (2) the outcome when treatment is positive (shown in yellow). Right: the ContinuousCausalModel setting. Here, the model can recover the entire dose–response curve (green curve) and identify the optimal dosage (orange diamond).

Specifically, we demonstrate that continuous causal inference models can be used to estimate individualized dose–response curves for physiotherapy intensity, enabling the identification of optimal treatment durations that improve functional independence. We focus on the effect of physiotherapy on activities of daily living (ADL), a validated measure of functional independence encompassing mobility and self-care (20), using Community Care and Home Care records from the Canadian InterRAI dataset (21). The resulting dose–response curves provide clinically interpretable insights into how therapy intensity affects functional outcomes. To further strengthen the clinical credibility of our estimates, we introduce a novel validation procedure based on proxy counterfactual matching. In summary, this paper makes the following three key contributions:

1. We show that continuous dose–response causal models yield more accurate predictions and more actionable insights than binary treatment-effect models for NPIs.
2. We demonstrate that recently developed continuous-treatment causal inference methods outperform traditional approaches, such as generalized propensity score models, in estimating the effect of physiotherapy on activities of daily living by more effectively capturing real-world variation in treatment intensity.
3. We introduce a novel validation procedure for approximating unobserved counterfactual outcomes, enabling more rigorous model evaluation and increasing clinical confidence in causal inference–based decision support.

## 2 Materials and methods

All analyses were performed in a secure virtualized computing environment running Microsoft Windows Server 2016 Standard. The system was equipped with an Intel® Xeon® CPU E5-2640 @ 2.50 GHz, 12 GB RAM, and a Microsoft Hyper-V virtual GPU for display.

### 2.1 Ethics approval and consent to participate

This study uses de-identified data obtained from the InterRAI Canada Data Server at the University of Waterloo, one of the designated international InterRAI data centers. The InterRAI assessment systems are standardized instruments used internationally to evaluate the needs and functioning of individuals receiving health and social services (21). All data are fully de-identified prior to researcher access and are subject to strict privacy, confidentiality, and security protocols. Data access requires completion of the Tri-Council Policy Statement: Ethical Conduct for Research Involving Humans (TCPS 2) course, execution of a confidentiality agreement, and approval of a formal data access request. The use of these de-identified data for the present study was reviewed and approved by virtue of completing the Ethical Conduct course, the InterRAI non-disclosure agreement and approval by the InterRAI organization.

### 2.2 Problem setup

Our objective is to estimate the causal effect of physiotherapy intensity T on activities of daily living (ADL; outcome Y) measured at follow-up, conditional on baseline individual characteristics X. We consider observational data in which each individual i is characterized by a vector of baseline covariates $x_{i}$, the dose of physiotherapy received $t_{i}$, and the corresponding follow-up ADL outcome $y_{i}$, viewed as realizations of the random variables X, T, and Y, respectively. Physiotherapy exposure is quantified as the total number of minutes of therapy received during the seven days preceding the outcome assessment.

More generally, a continuous treatment is characterized by both its type (the intervention provided) and its dose (the level or intensity of that intervention) (18). In this study, we restrict attention to a single treatment type: physiotherapy. The causal estimand of interest is the *personalized dose–response function*, which describes, for each individual, the expected ADL outcome as a function of physiotherapy intensity (illustrated by the green curve in Figure 1). This function provides a continuous characterization of how outcomes vary across the range of feasible treatment intensities.

To draw causal conclusions from observational data, we rely on standard assumptions from the causal inference literature (22). First, we assume *unconfoundedness*, such that all variables jointly influencing physiotherapy dose and ADL outcomes are captured in the observed baseline covariates X. Second, we assume *positivity*, meaning that individuals with similar covariate profiles have a nonzero probability of receiving a range of physiotherapy doses; in practice, this assumption is addressed by restricting the analysis to regions of sufficient treatment overlap. Finally, we assume *consistency*, whereby the observed outcome corresponds to the potential outcome under the dose actually received, and *no interference* between individuals, consistent with the Stable Unit Treatment Value Assumption (SUTVA).

### 2.3 Data and study design

Our training and validation datasets are derived from Canadian community care records collected in Ontario using InterRAI assessment instruments, specifically the Community Health Assessment and Home Care Assessment systems. These observational data are used for model training and internal (proxy counterfactual) validation, while an independent set of Canadian InterRAI Home Care data is reserved for external validation. The datasets contain repeated assessments for each individual, typically conducted at intervals of approximately three months.

For each individual, baseline covariates and ADL scores are extracted from a designated baseline assessment. Treatment values used for prediction are also taken from this baseline assessment. The ADL score measured at the subsequent follow-up assessment serves as the outcome variable for model training and evaluation. We denote these measures as ${\text{ADL}}_{\text{baseline}}$ and ${\text{ADL}}_{\text{\textbackslash{},follow-up}}$, respectively. Although the source data contain repeated assessments, the analytical dataset includes only one baseline–follow-up pair per individual; therefore, within-subject correlation does not arise.

#### 2.3.1 Cohort selection: inclusion and exclusion criteria

Several exclusion criteria were applied to construct a clinically meaningful cohort. Specifically, we exclude individuals who, based on clinical guidelines and expert judgment, would not typically receive PT. This includes individuals experiencing severe pain (encoded as J2A = 3), those in the end stage of disease (encoded as J5C = 1), individuals receiving palliative care (encoded as I1AO = 1), and individuals who were comatose at assessment (encoded as B1 = 1). Retaining such individuals would violate the assumption that individuals with similar covariate profiles could feasibly receive a range of physiotherapy doses. All individuals not excluded on the basis of these criteria were included to preserve adequate sample size and to allow model evaluation across a broad and clinically representative population.

#### 2.3.2 Population characteristics

The study population consists of older adults receiving Community Care and Home Care services in Ontario, Canada, who were assessed using the InterRAI Community Health Assessment and Home Care instruments. Table 1 summarizes the key demographic and clinical characteristics of the training, proxy counterfactual validation, and factual validation datasets used in this study. Across datasets, individuals were of advanced age and exhibited substantial variability in baseline functional status and physiotherapy exposure. Because suitable matches could not be identified uniformly across all patient profiles, the matched validation cohort represents a selected subset of the study population. As shown in Table 1, this cohort had substantially lower baseline ADL impairment than the training cohort. Consequently, the proxy counterfactual performance estimates primarily apply to this less impaired subset and may not generalize to the full training population, particularly to individuals with greater baseline impairment.

**Table 1:** Summary statistics for the training, proxy counterfactual validation, and factual validation datasets.

Dataset (*n*)	Variable	Mean	Std	Min	Max
Training (22,216)	Age (years)	83.1	10.5	49	112
	${\text{ADL}}_{\text{baseline}}$	12.7	7.7	0	28
	PT minutes	20.1	26.4	0	120
	Female, *n* (%)	15,911 (71.6%)			
Proxy counterfactual validation (2,138)	Age (years)	81.2	11.1	53	106
	${\text{ADL}}_{\text{baseline}}$	3.5	7.0	0	28
	PT minutes	23.2	31.0	0	260
	Female, *n* (%)	1,801 (84.2%)			
External validation (8,194)	Age (years)	83.0	10.3	51	106
	${\text{ADL}}_{\text{baseline}}$	12.6	7.8	0	28
	PT minutes	21.3	34.2	0	135
	Female, *n* (%)	5,998 (73.2%)			

Continuous variables are reported as mean and standard deviation (SD), with minimum (Min) and maximum (Max) values; categorical variables (e.g., gender) are reported as number (percentage).

#### 2.3.3 Covariate selection

The raw dataset contains 613 variables in total, including treatment and outcome measures. Because covariate selection is critical for causal inference and for the plausibility of the unconfoundedness assumption, substantial effort was devoted to identifying variables that capture clinically relevant sources of confounding. The selection process proceeded in two stages. First, an initial pre-selection step retained a broad set of candidate covariates based on clinical expertise and prior literature. Second, a refinement step removed covariates deemed less relevant, weakly predictive, or unlikely to influence either treatment assignment or outcomes. This process resulted in a final set of 14 covariates: (1) Gender, (2) Cognitive Performance Scale, (3) Flare-up, (4) Mode of nutritional intake, (5) Making oneself understood, (6) Socially appropriate behavior, (7) Bladder continence, (8) Bowel continence, (9) Foot problems, (10) Age at assessment, (11) Falls, (12) Depression Rating Scale, (13) Unsteady gait, and (14) Activities of Daily Living at baseline. A detailed description of the covariate selection procedure and the final set of included variables is provided in Supplementary Material S1.

#### 2.3.4 Outcome measure

We use the ADL Long Form (ADLL) scale as the primary outcome, as it provides a granular and clinically sensitive assessment of functional status. The ADLL ranges from 0 to 28, with higher scores indicating greater difficulty in performing activities of daily living, and is computed by summing scores across seven functional domains (23). The ADL Long Form Scale sums seven items covering activities such as bathing, dressing, eating, walking, and toileting. Each item is scored from 0 (independent or requiring setup help only) to 4 (total dependence), resulting in a total score ranging from 0 to 28. Although no validated thresholds for levels of dependency or clinically important change have been established, a two-point difference was considered potentially clinically relevant based on expert judgement. (24).

#### 2.3.5 Treatment variable

Physiotherapy exposure was operationalized as the total number of minutes of therapy received during the seven days preceding the baseline assessment (training distribution shown in Figure 2). Within the InterRAI system, physiotherapy exposure is recorded using the number of days on which therapy was administered during the preceding seven days (P1FA) and the total number of minutes delivered during that period (P1FB). We use total physiotherapy minutes because it provides a more granular measure of recent treatment intensity than the number of treatment days alone.

**Figure 2:**
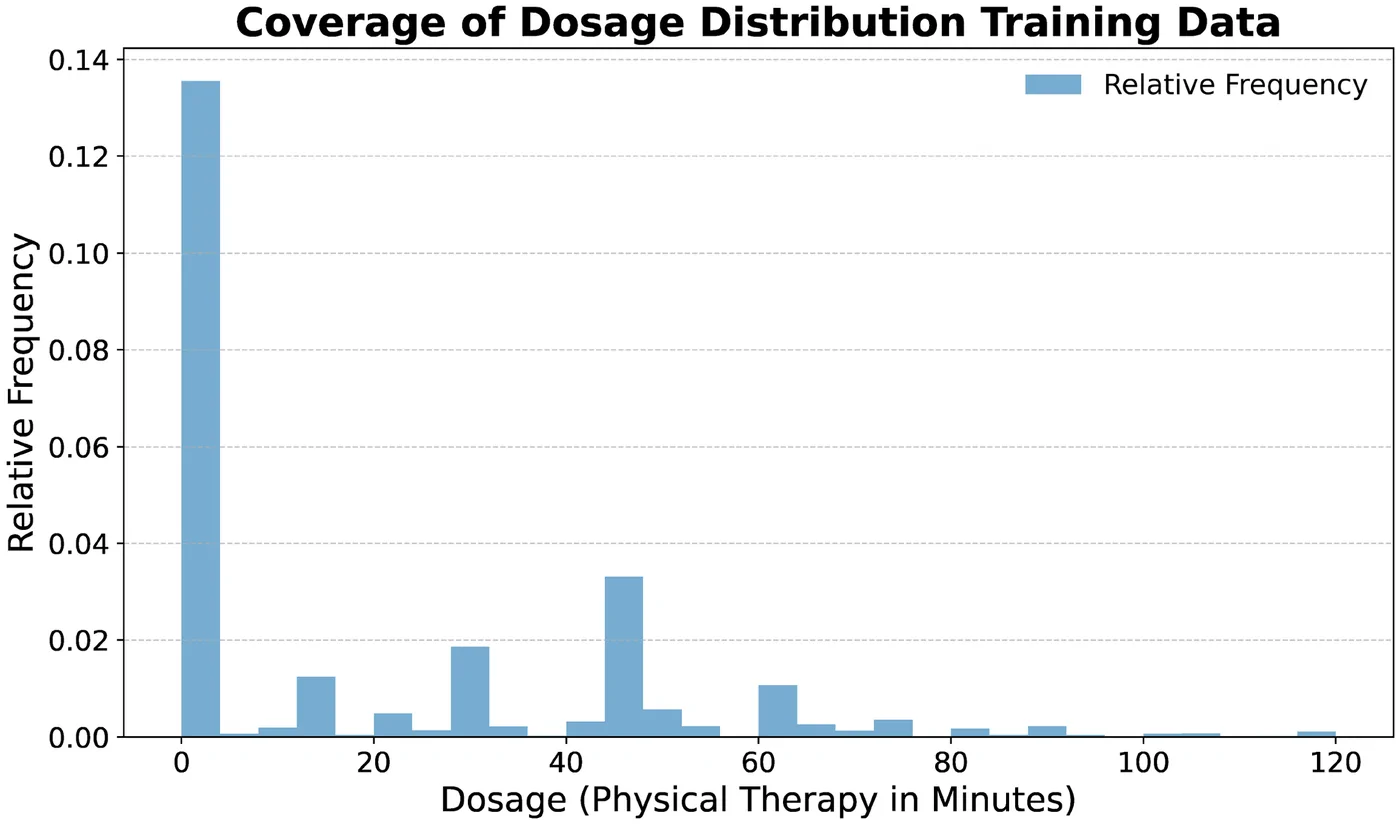
Distribution of physiotherapy doses (in minutes) in the training set. The majority of individuals receive little to no therapy, with substantially fewer observations at higher doses. This skewed distribution highlights the importance of accounting for limited support at upper dose ranges when estimating individualized dose–response curves.

The follow-up ADL assessment was generally conducted approximately three months later. Thus, the treatment variable represents a seven-day snapshot of recent physiotherapy exposure and is used as a proxy for continued weekly PT use between assessments. This assumption is supported by an internal analysis of the Canadian dataset, in which only 7.7% of PT recipients discontinued PT between assessments. Although less precise than repeated measurements throughout the follow-up period, this measure still captures variation in PT intensity and allows its association with ADL approximately three months later to be estimated. Nevertheless, interpretation of this measure as an indicator of usual treatment intensity assumes that the recorded week is reasonably representative of the individual’s broader treatment pattern.

### 2.4 Machine learning models

We employ an attention-based continuous counterfactual neural network based on the model proposed by Kazemi and Ester (25) to evaluate the performance of continuous-treatment causal models in estimating the effect of physiotherapy duration (in minutes) on functional independence. We call our implementation CausalPhysioNet, and it is essentially a neural network architecture specifically designed for continuous treatment effect estimation of physiotherapy. A more detailed discussion on our approach can be found in Supplementary Material 2.

To benchmark our approach, we compare its predictions against several established causal inference methods and baseline models. Specifically, we include the generalized propensity score (GPS) approach (26), which extends classical propensity score methods to settings with continuous treatments. We also evaluate the T-Learner (14), a meta-learning framework that combines treatment-specific models to estimate conditional treatment effects. Specifically, to instantiate we use a two-armed neural network. To implement the T-learner as a binary-treatment benchmark, we discretized PT exposure at 45 min. This pragmatic, data-driven threshold yielded greater separation in average outcomes than lower candidate thresholds. It is not intended as a clinical cut-off, and performance may depend on the chosen discretization. As a simple parametric baseline, we consider linear regression (LR), in which physiotherapy intensity is included as a covariate. In addition, we include the parametric $E_{\text{max}}$ dose–response model (27), which is widely used in clinical and pharmacological studies to represent monotonic continuous dose–response relationships. All baseline methods are implemented using standard Python libraries.

#### 2.4.1 Model training & hyperparameter tuning

Hyperparameters for all models were selected using randomized or grid search with 5-fold cross-validation on a factual validation set constructed from observed data. For baseline models implemented using scikit-learn, including linear regression (LR) and the X-Learner, standard hyperparameters were tuned over commonly used ranges. For continuous-treatment and dose–response models, including the generalized propensity score (GPS), the parametric $E_{\text{max}}$ model, and CausalPhysioNet, hyperparameters were tuned over search spaces commonly adopted in the literature (28). Additional model-specific parameters—such as clinically plausible dose–response constraints for the $E_{\text{max}}$ model and regularization terms for CausalPhysioNet—were selected based on validation performance. Complete hyperparameter search spaces and tuning details are provided in Supplementary Material 3.

### 2.5 Model evaluation methodology

Evaluating causal models is inherently challenging because counterfactual outcomes are not directly observable (29). We therefore employ multiple complementary evaluation strategies to assess model performance. Specifically, we conduct a comparative evaluation of the proposed Varying Coefficient Network against baseline methods using both factual and proxy counterfactual validation. The objective is to assess whether explicitly modeling continuous treatment effects yields advantages over simpler causal and non-causal approaches.

Factual validation is conducted using observed individual outcomes in a held-out validation cohort and evaluates model performance under physiotherapy doses that were actually delivered in routine care and recorded by Canadian healthcare professionals. This assessment quantifies how accurately each model predicts realized functional outcomes for individuals receiving different levels of physiotherapy. In addition, we perform external validation using an independent Canadian Home Care dataset, which differs from the Community Care training data in both patient case-mix and care setting.

Proxy counterfactual evaluation assesses model predictions under alternative physiotherapy doses that were not observed for a given individual. Because such counterfactual outcomes are inherently unobservable, we approximate them using a matched-pairs strategy, illustrated in Figure 3. Specifically, the data are partitioned into a model training cohort and a candidate matching cohort. For each individual in the training cohort, we identify a clinically similar individual—matched on baseline covariates—who received a different physiotherapy dose. This procedure yields a matched validation cohort that represents plausible alternative treatment scenarios for proxy counterfactual assessment. Because each individual can receive only one physiotherapy dose at a given time, the corresponding outcomes under alternative doses are inherently unobservable. Therefore, individual-level counterfactual predictions cannot be validated against a real-world gold standard. Instead, this procedure evaluates model predictions against outcomes observed in clinically similar individuals who received different physiotherapy doses. These matched outcomes should be interpreted as assumption-dependent proxies whose credibility relies on adequate covariate balance, treatment overlap, and the absence of important unmeasured confounding.

**Figure 3:**
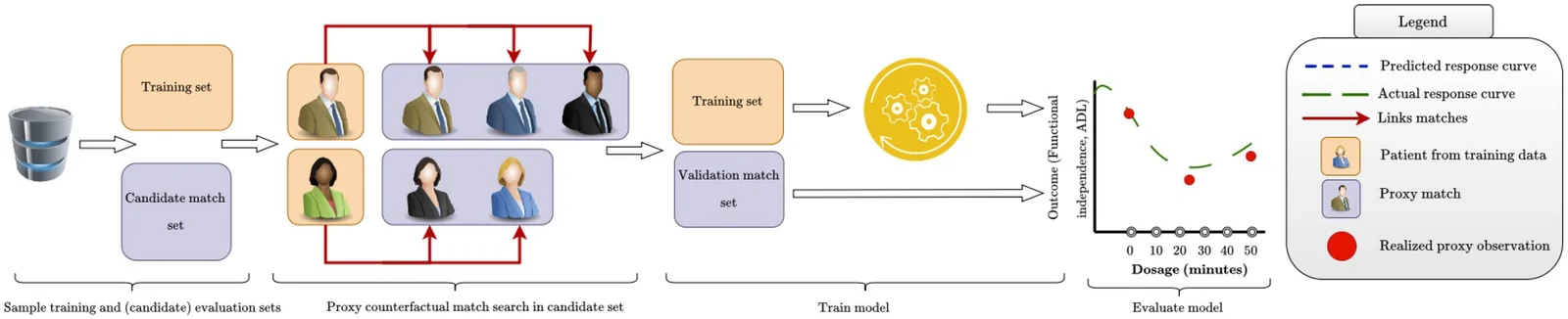
Overview of the proxy counterfactual evaluation pipeline for continuous dose–response modeling. Individual records are first partitioned into a training cohort and a candidate matching cohort. The training cohort is used to fit models that estimate individualized outcome trajectories as a function of physiotherapy dose. For proxy counterfactual evaluation, each individual in the training cohort is paired with a clinically similar individual from the candidate cohort who received a different physiotherapy dose, yielding a validation match set that serves as a proxy for unobserved counterfactual outcomes. Model-predicted outcomes at alternative doses are then compared against the observed outcomes of matched individuals to assess proxy counterfactual predictive performance.

To avoid over-representation of common individual profiles or treatment intensities, duplicated matches are removed from the proxy counterfactual validation set. Model predictions for unobserved treatment doses are then compared against the observed outcomes of matched individuals. For example, for an 85-year-old female who received 45 min of physiotherapy, the model’s predicted outcome under a lower dose (e.g., 15 min) is compared with the observed outcome of a clinically similar individual who received that lower dose. Although this approach does not recover true counterfactual outcomes, it provides a clinically grounded and pragmatic approximation for evaluating causal models using observational healthcare data.

Model performance is quantified using the mean absolute error (MAE) for factual evaluation, defined as

$$\text{MAE}=\frac{1}{n}\sum_{i=1}^{n}|y_{i}-{\hat{y}}_{i}|,$$

and the root mean squared error (RMSE) for counterfactual evaluation, defined as

$$\text{RMSE}=\sqrt{\frac{1}{n}\sum_{i=1}^{n}{\left(y_{i}-{\hat{y}}_{i}\right)}^{2}},$$

where ${\hat{y}}_{i}$ denotes the model-predicted outcome for individual i.

## 3 Results

We evaluate whether continuous causal models can recover clinically meaningful dose–response relationships between physiotherapy time intensity and functional independence. We first report quantitative performance under factual, proxy counterfactual, and external validation using the evaluation framework described in Subsection 2.5. We then present representative individualized dose–response curves and discuss their clinical interpretation. Finally, we examine the contribution of baseline covariates through an ablation analysis (Subsection 3.3).

### 3.1 Baseline evaluation

#### 3.1.1 Factual evaluation results

Table 2 (columns 1–2) reports performance on the factual validation dataset in terms of RMSE and MAE for the ADL outcome. Among the evaluated methods, the X-Learner achieves the lowest factual RMSE (3.69±0.69), while CausalPhysioNet attains the lowest MAE (2.05±0.81) alongside a comparably low RMSE (3.79±0.77). However, the difference between the two models was not statistically significant (p≥0.05), indicating comparable factual RMSE performance. CausalPhysioNet achieved the lowest factual MAE (2.05 ± 0.81). Linear regression also performs competitively, with factual errors of 4.09±1.27 in RMSE and 3.09±0.83 in MAE. In contrast, the GPS and parametric $E_{\text{max}}$ models exhibit substantially higher errors across both metrics. From a clinical perspective, these results indicate that adopting a flexible dose–response model such as CausalPhysioNet does not compromise predictive accuracy on observed outcomes, supporting its suitability for downstream counterfactual analysis and clinical decision-support applications.

**Table 2:** Mean ± standard deviation of RMSE and MAE across five runs for each method under factual, proxy counterfactual, and external validation.

Method	Factual		Proxy counterfactual		External	
	RMSE	MAE	RMSE	MAE	RMSE	MAE
GPS	7.64 ± 1.10	6.31 ± 0.75	10.61 ± 0.94	10.65 ± 1.39	8.13 ± 1.01	6.48 ± 1.25
X-Learner	**3.69 ± 0.69**	3.04 ± 0.48	4.19 ± 0.76	3.88 ± 0.84	5.10 ± 0.61	4.88 ± 0.72
LR	4.09 ± 1.27	3.09 ± 0.83	6.49 ± 1.26	6.16 ± 0.94	3.93 ± 0.69	3.67 ± 0.60
$E_{\text{max}}$	7.12 ± 1.10	6.72 ± 0.95	8.12 ± 1.67	7.12 ± 1.05	6.72 ± 0.71	6.52 ± 0.83
CausalPhysioNet	**3.79 ± 0.77**	**2.05 ± 0.81**	**3.89 ± 0.58**	**2.49 ± 0.72**	**3.85 ± 0.67**	**2.29 ± 0.40**

Bold values indicate the best-performing model or models with statistically comparable performance (p>0.05; paired t-test).

#### 3.1.2 Proxy counterfactual evaluation results

Proxy counterfactual evaluation results, reported in columns 3–4 of Table 2, show that CausalPhysioNet achieves the lowest prediction error among all evaluated methods, with a RMSE of 3.89±0.58 and a MAE of 2.49±0.72. The X-Learner also performs well in this setting, yielding a RMSE of 4.19±0.76 and a MAE of 3.88±0.84. Linear regression exhibits substantially higher proxy counterfactual error, with values of 6.49±1.26 for RMSE and 6.16±0.94 for MAE. In contrast, the GPS and parametric $E_{\text{max}}$ models show markedly larger errors across both metrics, indicating limited suitability for counterfactual outcome estimation in this setting.

Overall, these results highlight the advantage of models that explicitly encode continuous treatment effects when estimating counterfactual outcomes. From a clinical perspective, improved proxy counterfactual accuracy is critical for informing treatment planning decisions that involve adjusting physiotherapy intensity beyond previously observed regimens.^2^ Furthermore, the results should be interpreted in light of differences between the proxy counterfactual validation and training populations. In particular, the matched validation cohort had substantially lower baseline ADL impairment. The reported proxy counterfactual errors therefore primarily reflect model performance in this less impaired matched subset and may not generalize to the full training population, particularly to individuals with greater baseline impairment. Still, a trade-off exists between model expressiveness and robustness: by leveraging complex interactions among patient-specific covariates to generate individualized predictions, CausalPhysioNet becomes more sensitive to missing input data than simpler parametric models such as $E_{\text{max}}$.

#### 3.1.3 External validation results

Finally, we assess model generalization by externally validating models trained in the Community Care setting on an independent Home Care dataset. Results are reported in columns 5–6 of Table 2. Overall, most methods exhibit performance comparable to that observed under factual evaluation, suggesting robustness to shifts in clinical context and data source. CausalPhysioNet achieves the strongest external performance, with the lowest MAE (2.29±0.40) and a low RMSE (3.85±0.67). Linear regression also generalizes well, yielding external errors of 3.93±0.69 in RMSE and 3.67±0.60 in MAE. The X-Learner shows moderate degradation in performance relative to factual evaluation, while the GPS and parametric $E_{\text{max}}$ models continue to exhibit substantially higher errors across both metrics. Collectively, these findings indicate that models capable of capturing flexible dose–response relationships, such as CausalPhysioNet, maintain predictive accuracy across care settings, supporting their potential applicability in real-world clinical decision-support scenarios.

### 3.2 Dose-response curves analysis

To provide intuition for the outputs of a continuous causal effect model, we examine four representative dose–response curves generated by the CausalPhysioNet model. These curves, shown in Figure 4, depict individualized dose–response relationships for four individuals that a clinician might encounter in a real-world decision-support dashboard.^3^ Each curve corresponds to a distinct response profile.

**Figure 4:**
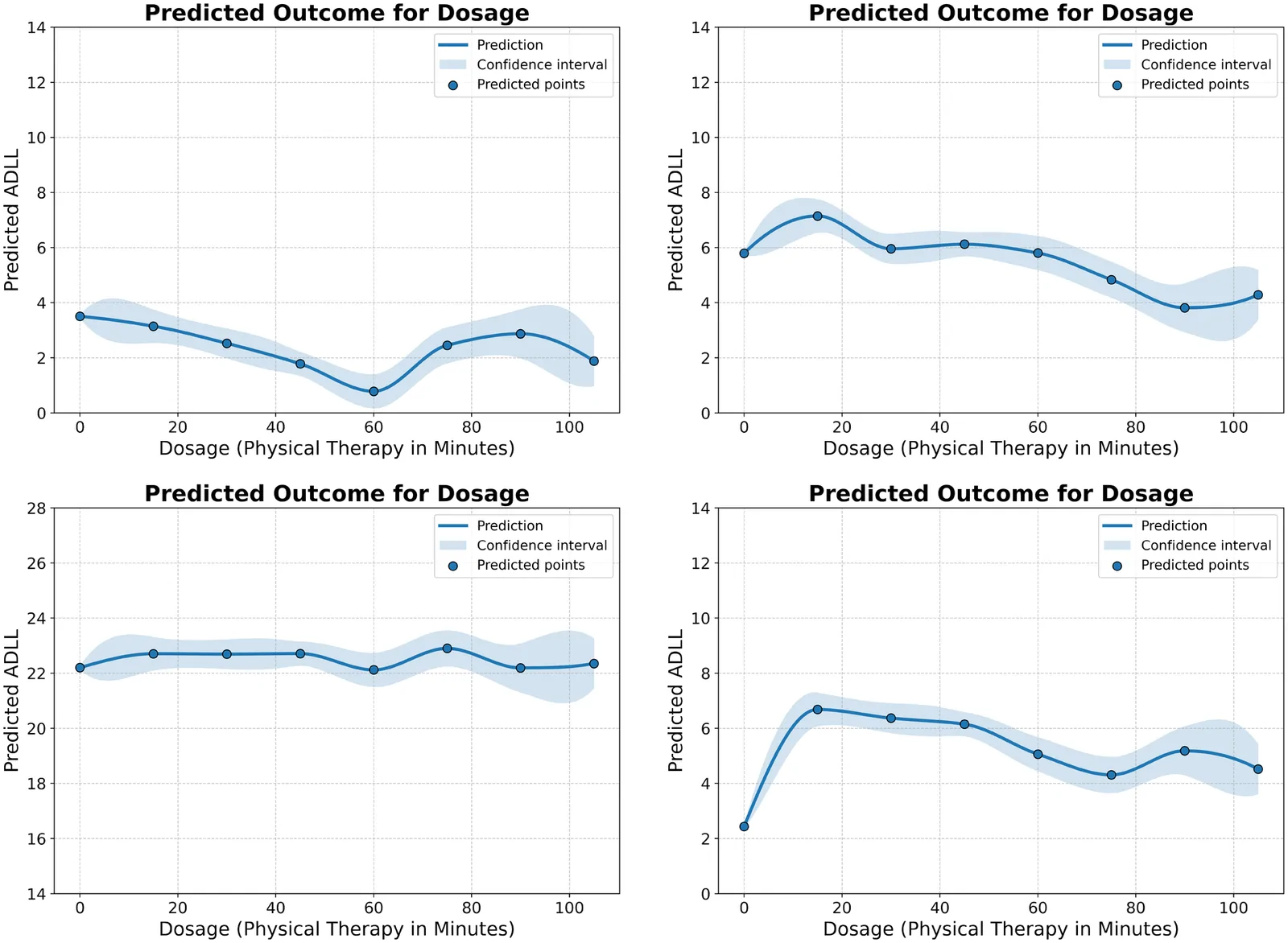
Predicted individualized dose–response curves generated by CausalPhysioNet for four individuals from the matched proxy counterfactual validation set. The x-axis shows physiotherapy minutes and the y-axis shows predicted ADL at follow-up, with lower ADL indicating better functional independence. Solid lines represent model predictions and shaded regions represent 95% uncertainty bands. The top-row individuals show predicted improvement with increasing physiotherapy intensity; the top-left curve has an estimated minimum at approximately 60 min. The bottom-left curve shows little predicted benefit, whereas the bottom-right curve shows predicted deterioration at higher intensities. Because empirical support is limited at higher physiotherapy doses, curve features and minima in these regions should be interpreted as exploratory rather than as definitive treatment recommendations.

The two curves in the top row represent individuals for whom the model predicts improved ADL outcomes with physiotherapy. For the individual shown in the top-left panel, the model predicted a reduction of approximately 2.2 points on the ADL Long Form Scale at around 60 min of physiotherapy relative to no treatment. Although a validated minimal clinically important difference has not been established for this scale, a two-point change represents a tangible change in functional support needs based on the scale’s item-level assistance categories and was therefore considered clinically relevant by experts within the author team. In contrast, the top-right individual shows meaningful improvement only at higher doses (around 90 min), highlighting substantial inter-individual variation in optimal treatment intensity.

The bottom row illustrates alternative response patterns. The bottom-left individual shows no observable treatment effect, with a nearly flat curve suggesting limited therapeutic benefit; in such cases, a clinician might reasonably opt against prescribing physiotherapy. The bottom-right individual exhibits a negative response pattern, in which increasing therapy intensity is associated with worsening ADL scores. Such patterns may arise when physiotherapy is delivered at excessive intensity or duration—an “overdosed” intervention (30)—potentially leading to adverse effects such as pain or stiffness that impair functional outcomes (31, 32).

To assess broader response patterns, we analyzed a random sample of n=134 individuals from the proxy-counterfactual validation set. Downward trends—indicating improved ADL with increasing physiotherapy intensity—were most common (43.7%), followed by flat or no-effect patterns (27.2%). Upward trends, corresponding to declining functional outcomes with increasing treatment intensity, occurred in 13.6% of cases, while the remaining 15.5% exhibited noisy or mixed responses. Overall, beneficial response patterns predominated, with adverse or heterogeneous patterns occurring less frequently.

Although a comprehensive subgroup analysis lies beyond the scope of this work, one notable association emerged: a history of falls was strongly associated with noisy or unfavorable dose–response patterns. Individuals with prior falls accounted for approximately 69% of noisy trends and 67% of upward trends, compared with 31% and 33%, respectively, among individuals without a fall history.

### 3.3 Sensitivity analysis

Finally, we examine the robustness of the evaluated models to violations of the unconfoundedness assumption. Most causal inference methods rely on the assumption that all relevant confounders are observed; however, this condition is difficult to satisfy in clinical practice, where important variables may be unmeasured or inconsistently recorded. To assess robustness to such violations, we retrained each model multiple times while randomly omitting one, two, or three covariates, and quantified the resulting degradation in performance (Δ) relative to the full-covariate baseline (Table 3).

**Table 3:** Absolute performance degradation (Δ) over 5 runs, measured in terms of RMSE, when randomly dropping one, two, or three covariates.

Method	Covariate drop robustness		
	$\Delta_{\text{1 Covariate}}$	$\Delta_{\text{2 Covariates}}$	$\Delta_{\text{3 Covariates}}$
GPS	0.36 ± 0.11	0.33 ± 0.19	0.39 ± 0.22
LR	0.04 ± 0.03	0.11 ± 0.09	0.48 ± 0.12
X-Learner	**0.03 ± 0.05**	0.42 ± 0.13	0.88 ± 0.43
$E_{\text{max}}$	0.02 ± 0.02	**0.06 ± 0.02**	**0.17 ± 0.04**
CausalPhysioNet	0.79 ± 0.11	0.78 ± 0.13	1.46 ± 0.21

Lower values indicate greater robustness. Bold values indicate the lowest mean per column (p≤0.05; Welch’s paired t-test), comparing the best-performing model with the second-best-performing model in each column.

The results reveal substantial heterogeneity in robustness across methods. CausalPhysioNet exhibits the largest performance degradation under covariate omission, with Δ=0.79±0.11 when a single covariate is removed, increasing to Δ=1.46±0.21 when three covariates are omitted. This pattern indicates a strong dependence on comprehensive covariate information. In contrast, the parametric E${}_{\text{max}}$ model remains comparatively stable across all settings, with degradations of 0.02±0.02, 0.06±0.02, and 0.17±0.04 for one, two, and three omitted covariates, respectively. Linear regression similarly shows limited sensitivity when one or two covariates are removed, but experiences a more pronounced increase in degradation when three covariates are omitted (Δ=0.48±0.12). The X-Learner demonstrates low sensitivity to the removal of a single covariate (Δ=0.03±0.05), but degrades more substantially as additional covariates are omitted. Lastly, because InterRAI records physiotherapy only for the seven days preceding baseline, cumulative exposure over the three-month follow-up is unavailable. Longer longitudinal treatment records with session-level timing and duration are needed to assess cumulative and sustained effects.

Overall, these findings indicate that while all methods are affected by omitted-variable bias, more expressive dose–response models such as CausalPhysioNet may be particularly sensitive to covariate misspecification. This underscores an important trade-off between model flexibility and robustness in applied clinical settings, where covariate completeness cannot always be guaranteed.

## 4 Discussion

In this study, we investigated the potential of continuous causal machine learning to model dose–response relationships for physiotherapy in older adults, with a particular focus on quantifying the effect of physiotherapy intensity on activities of daily living. Although non-pharmacological interventions are increasingly recognized for their role in improving functional outcomes and quality of life, their optimal dosing remains poorly understood due to substantial inter-individual heterogeneity and the lack of robust analytical frameworks. Our findings demonstrate that continuous causal machine learning models can recover individualized dose–response curves from observational data, providing a data-driven foundation for more personalized and adaptive physiotherapy planning.

A key finding of this study is that specialized dose–response models can recover clinically meaningful relationships between physiotherapy intensity and functional outcomes that align with established rehabilitation practice. By capturing nonlinear dependencies between therapy dosage and ADLs, these models provide more granular insight into inter-patient variability than traditional approaches. This capability holds promise for tailoring interventions to the specific needs of older adults, with the potential to improve treatment effectiveness while supporting more efficient use of clinical resources. Moreover, individualized dose–response estimates may reduce reliance on trial-and-error dosing strategies by offering clinicians evidence-based guidance on how adjustments in therapy intensity are likely to influence functional outcomes.

Several limitations warrant consideration. Although our framework enables a systematic approximation of counterfactual outcomes, proxy counterfactual validation remains inherently challenging in observational settings. The matched outcomes used in the proxy counterfactual evaluation are not true counterfactual outcomes. Because the missing potential outcome is unobserved for every individual, it is not possible to determine directly how closely a matched outcome approximates that individual’s true response under an alternative dose. The results should therefore be interpreted as a pragmatic, assumption-dependent assessment of model behaviour rather than as evidence of ground-truth counterfactual accuracy. The surrogate matching procedure employed in this study depends on the quality and coverage of the available data and may degrade in the presence of sparse, noisy, or systematically biased observations. In addition, baseline ADL values in the proxy counterfactual validation dataset differ substantially from those in the training and external validation sets. Moreover, while our approach offers a practical alternative to randomized controlled trials (RCTs) for studying continuous interventions, it does not replicate the causal guarantees provided by randomization. Validation across diverse clinical populations, care settings, and healthcare systems will therefore be necessary to assess the robustness and generalizability of these findings.

Future work should extend these methods to a broader range of outcomes and non-pharmacological interventions such as cognitive behavioral therapy, nutritional programs, or social engagement initiatives to better understand how different interventions contribute to individual well-being. Importantly, the proposed framework can be adapted to study combined or sequential interventions, enabling analysis of how multiple therapies interact to influence functional outcomes. Beyond physiotherapy and ADLs, applying continuous causal models to additional intervention–outcome pairs could substantially expand the scope of personalized care research. The covariate-drop analysis identifies an important direction for future work: developing missingness-aware architectures and imputation strategies that preserve CausalPhysioNet’s ability to generate individualized predictions while improving its robustness to incomplete clinical data. Any future clinical implementation should additionally use a prespecified, clinically justified input set and include safeguards to ensure that essential patient information is available before predictions are generated.

Another important direction for future research is to identify the conditions under which dose–response models perform optimally. This includes examining how baseline individual characteristics, comorbidities, environmental factors, and care pathways shape estimated dose–response relationships. Subgroup analyses, although beyond the scope of the present study, may help identify which individuals benefit most from specific dosing strategies and why, underscoring the need for close collaboration between methodological and clinical experts. For instance, a history of falls may reflect greater frailty, impaired balance, or broader clinical complexity, which may partly explain the more heterogeneous or unfavorable dose–response patterns observed in this subgroup. Furthermore, future work could explore combining our approach with stacking methods such as SuperLearner to improve model stability under noisy or incomplete covariate information. However, such ensemble methods would not by themselves address bias arising from unmeasured confounding.

Finally, integrating continuous dose–response models into real-world clinical decision-support systems represents a promising long-term objective. Achieving this goal will require careful attention to model robustness, interpretability, and safety, as well as explicit consideration of ethical and equity-related concerns. Addressing these challenges through sustained interdisciplinary collaboration will be essential for building clinician trust and ensuring that the potential benefits of personalized dosing strategies are realized equitably across patient populations.

## Data Availability

The data analyzed in this study is subject to the following licenses/restrictions: Only InterRAI Fellows and students of InterRAI fellows have access to the data after completing Ethical Research Module Course, signing of a confidentiality agreement and a non-disclosure agreement. Requests to access these datasets should be directed to micaela.jantzi@uwaterloo.ca.

## Footnotes

^1^We follow a standard definition used by clinicians (e.g., https://world.physio/resources/what-is-physiotherapy, assessed 21-01-2026).

^2^While proxy counterfactual validation provides a controlled proxy for assessing model behavior under unobserved treatment intensities, true counterfactual outcomes are inherently unobservable in observational data. Accordingly, these results should be interpreted as indicative rather than definitive evidence of counterfactual accuracy. An additional support analysis is provided in Supplementary Material 4.

^3^On the ADL scale, values closer to 0 indicate higher functional independence.
