# Supplementary material for: Personalized physiotherapy effect modeling with continuous causal machine learning
Source: Front Digit Health. 2026 Oct 27;8:1793661. doi: 10.3389/fdgth.2026.1793661 (PMC13748423; doi:10.3389/fdgth.2026.1793661)
Supplement: Supplementary file 1 [file Datasheet1.pdf]

# Supplementary Material

## 1 COVARIATE SELECTION PROCESS

| Abbreviation | Name | Purpose | Range |
|---|---|---|---|
| AA2 | Gender | Biological sex of the individual | Male / Female |
| CPS | Cognitive Performance Scale | Reflects cognition, based on memory, consciousness, and executive function | 0–6 |
| J5B | Flare-up | Indicates whether the individual has experienced a flare-up of a chronic condition | Yes / No |
| K5B | Mode of nutritional intake | Describes how nutrition is administered | Oral / Tube-fed / Parenteral |
| D1 | Making oneself understood | Assesses ability to communicate needs and ideas | 0–4 |
| E4DA | Socially appropriate behaviour | Evaluates appropriateness of social behaviour | 0–3 |
| H1B | Bladder continence | Measures bladder control | 0–4 |
| H1A | Bowel continence | Measures bowel control | 0–4 |
| M6A | Foot problems | Presence of foot issues affecting mobility or comfort | Yes / No |
| Age | Age at assessment | Age in years at time of assessment | Continuous (years) |
| J4B | Falls | Indicates whether the individual has had one or more falls | Yes / No |
| DRS | Depression Rating Scale | Measures signs and symptoms of depression | 0–14 |
| J1N | Unsteady gait | Indicates whether unsteady gait is observed | Yes / No |
| P1PCB | physiotherapy – minutes | Number of minutes of physiotherapy received | Continuous (minutes/week) |
| ADL base | Activities of Daily Living at Baseline | Baseline measure of ADL performance | 0–28 |

**Table S1.** Description of final selection of covariates and their ranges. Purpose indicates the purpose of measurement. Range indicates the possible values or scales.

**Initial Pre-selection.** Based on expert input, we initially identified 38 covariates for inclusion in the analysis. These variables were selected from the clinical literature and expert opinion as factors likely to influence either the amount of physiotherapy received (in minutes) or the ADL outcome. The pre-selection process was conducted by a team of three clinical researchers under the supervision of a practitioner–researcher in geriatric medicine. The resulting list was subsequently reviewed and refined through discussion with a panel consisting of five clinicians and clinical researchers, as well as two machine learning analysts with experience in clinical applications. The initial list of covariates used in this study are defined as follows:

- **Demographics:** gender, age at assessment, resident identifier
- **Cognition and mental health:** Cognitive Performance Scale (0–6), Depression Rating Scale, presence of delusions
- **Clinical conditions:** chest pain or discomfort, constipation, fever, aspiration, recent flare-ups, end-stage disease indicators, dizziness, unsteady gait, falls (past 31–180 days)
- **Treatments and clinical supports:** chemotherapy, oxygen therapy, suctioning, tracheostomy care, blood transfusion, ventilator support, radiation therapy
- **Restraints and nutrition:** use of full bed rails, chair restraints preventing rising, mode of nutritional intake (parenteral/IV fluids, feeding tube, mechanically altered diet)
- **Functional and behavioral measures:** vision in adequate light, frequency of wandering
- **Continence and related devices:** bladder continence, bowel continence, use of urinary devices (condom catheter, indwelling catheter, intermittent catheter)
- **Skin integrity and mobility limitations:** foot problems (infection, open lesions, dressings), pressure ulcers (severity 0–4)

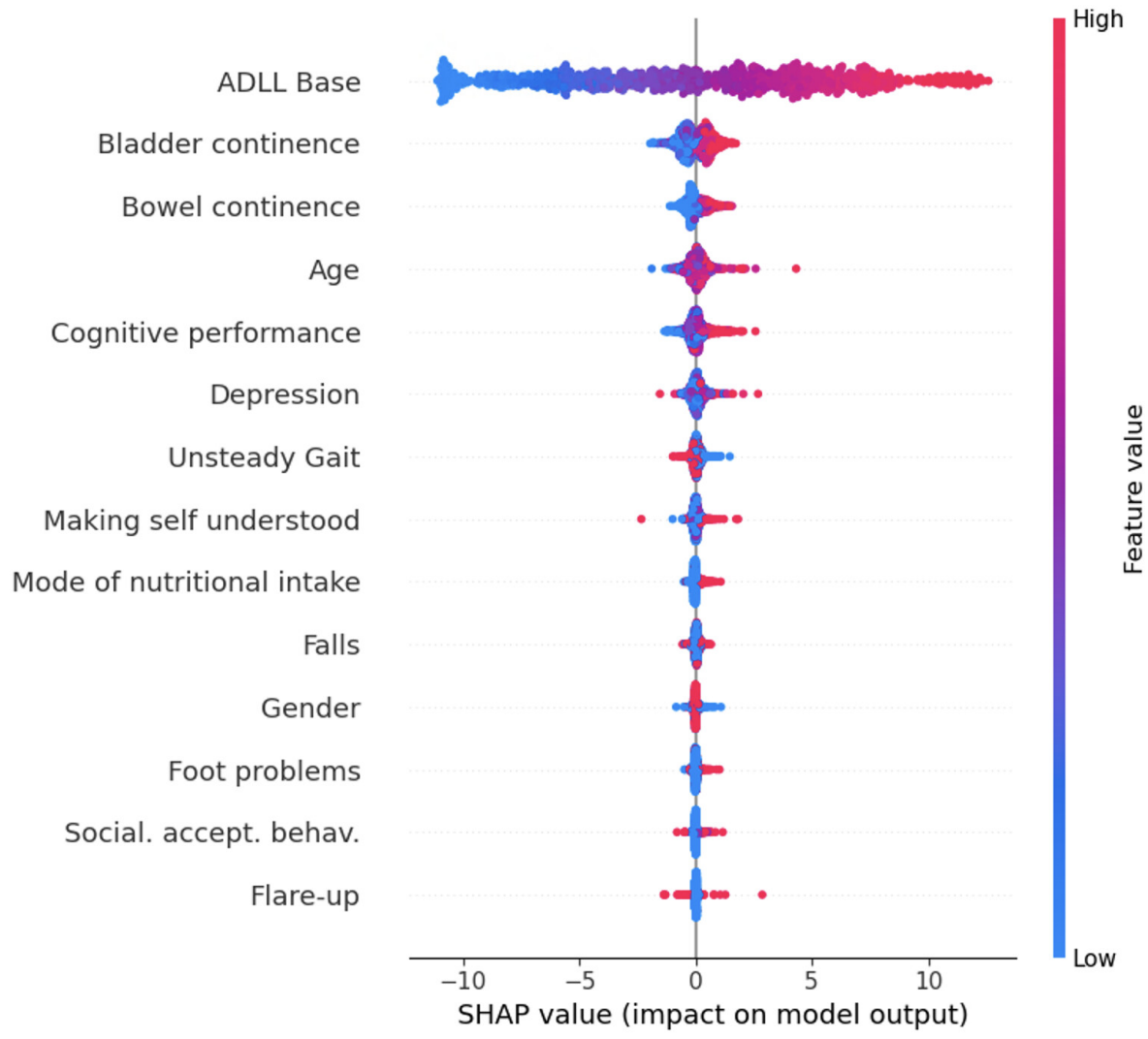


**Figure S1.** SHAP summary plot showing feature importance and direction of effect on the model output. Each point represents an observation in the dataset, with the horizontal axis indicating the SHAP value (the contribution of the feature to the prediction). Features are ordered by their overall importance, with those at the top contributing most strongly to the model's decisions. The color scale represents the feature value (red = high, blue = low). For example, higher baseline ADL scores (ADL Base) are associated with increased predicted outcomes, while bladder and bowel continence, age, and cognitive performance also play key roles but with more balanced or heterogeneous effects. Features near the bottom (e.g., flare-ups, socially acceptable behavior) show smaller overall contributions.

**Final selection.** Following the pre-selection stage, we performed a final refinement of the covariate set guided by two considerations. First, exploratory data analysis indicated that several variables occurred at very low frequencies (e.g., oxygen therapy was recorded for approximately 2% of individuals after preprocessing), resulting in near-zero variance and limited predictive value. Second, retaining all 38 pre-selected covariates complicates post-hoc interpretation; focusing on a smaller, clinically relevant subset facilitates the identification of meaningful patterns, such as differential treatment effects across individual subgroups. Accordingly, variables were removed based on two criteria: low variance and limited importance. Covariates with minimal variability were excluded following standard machine-learning practice for removing near–zero-variance predictors (variance below 0.01) (**?**). Variable importance was assessed using Shapley value analysis (Figure S1), and covariates with Shapley values within the range $(-1, +1)$ were removed due to their negligible contribution to model predictions. The final list and specifications can be found in Table S1.

# 2 MODELING CONSIDERATIONS

## 2.1 CausalPhysioNet

CausalPhysioNet is designed to estimate individualized dose–response relationships, in which treatment effects depend not only on treatment assignment but also on dose intensity. The model builds on the ACFR architecture of Kazemi en Ester (2024), but replaces the adversarial learning component with a fully non-adversarial formulation that produces smooth dose–response curves across the full range of treatment levels while incorporating individual covariates. This allows the identification, for each individual, of the treatment dose expected to yield the greatest benefit, supporting precision dosing in clinical decision-making. To mitigate treatment assignment bias, we augment this architecture with an information-criterion regularizer based on the Cauchy–Schwarz divergence (Yu et al. 2024) The regularizer encourages statistical independence between the learned latent representation and the treatment variable, thereby reducing confounding effects (see Figure S2). This strategy is conceptually related to other regularization-based approaches for dose–response estimation, such as maximum mean discrepancy (MMD) (Smola et al. 2026), while avoiding adversarial optimization and improving interpretability. The model is trained by minimizing the following objective:

$$\mathcal{L} = \mathbb{E}\left[(y - \hat{y})^2\right] + \beta R_{\text{dim}} + \gamma R_{\text{treat}}, \tag{S1}$$

where the first term denotes the mean squared prediction error, $R_{\text{dim}}$ encourages a compact latent representation, and $R_{\text{treat}}$ penalizes dependence between the latent representation $z$ and treatment $t$ via the Cauchy–Schwarz divergence. In all experiments, we set $\beta = 0.05$ and $\gamma = 0.01$.

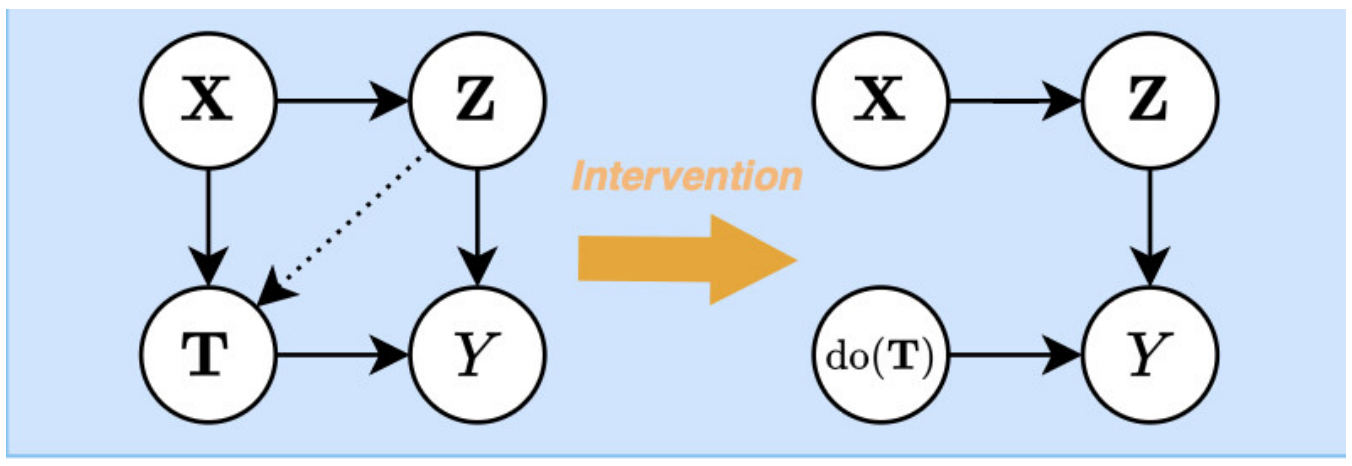


**Figure S2.** Causal schematic illustrating the estimation of the effect of physiotherapy (PT) on activities of daily living (ADL). On the left, $X$ denotes observed individual characteristics (e.g., age, comorbidities), $Z$ a latent representation learned from these features using the CausalPhysioNet model, $T$ the treatment received (amount of PT), and $Y$ the outcome (ADL). The dashed arrow indicates potential confounding, where $Z$ may be associated with both treatment assignment and outcomes. The intervention framework (right) shows the effect of actively setting treatment $T$ (denoted $do(T)$), which isolates the causal impact of PT on ADL while accounting for confounding.

## 2.2 Meta-learner

To ensure a fair comparison and avoid disadvantaging the meta-learner approach (Künzel et al. 2019), we instantiated it using a standard $T$-learner framework. We evaluated multiple base learners, including gradient boosting (GB), linear regression (LR), neural networks (NN), Lasso regression (Lasso), random forests (RF), and AdaBoost. As the meta-learner formulation requires a binary treatment indicator, we considered treatment thresholds of 15, 30, 45, and 60 minutes of physiotherapy. Among these configurations, the neural network base learner with a 45-minute threshold achieved the best performance and is therefore reported in the main results.

# 3 HYPERPARAMETER TUNING

Hyperparameter tuning was performed using randomized or grid search with 5-fold cross-validation, based on performance on the *factual* validation set (i.e., a standard hold-out set drawn from observed data). For the `sklearn` models, we conducted grid search over established parameter ranges. For the Random Forest Regressor (RFR), we tuned the number of trees ($n_{\text{estimators}} \in \{100, 200, 500\}$) and maximum tree depth ($\{3, 5, 10\}$). For linear regression (LR), we tuned the regularization strength ($C \in \{0.01, 0.1, 1, 10\}$). For GPS, MLP, and the $E_{\text{max}}$ model, hyperparameters were tuned over standard search spaces used in the literature (e.g. Pfob et al. (2022)), including learning rates ($\{10^{-4}, 10^{-3}, 10^{-2}\}$), L2 regularization strengths ($\{10^{-5}, 10^{-4}, 10^{-3}\}$), and hidden layer sizes ($\{32, 64, 128, 256\}$) for neural components. For the $E_{\text{max}}$ model specifically, we additionally searched over clinically plausible values of $E_{\max}$ (0–10), $ED_{50}$ (within the observed dose range), and the Hill coefficient ($\{0.5, 1, 2\}$) when applicable.

For CausalPhysioNet (implemented in PyTorch), we tuned hidden layer widths ($\{64, 128, 256\}$), learning rates ($\{10^{-4}, 10^{-3}\}$), batch sizes ($\{32, 64\}$), and dropout rates ($\{0.2, 0.5\}$). We also tuned the weight of a regularization term encouraging independence between the compressed feature representation and the treatment variable, using values in $\{0.1, 0.25, 0.5\}$.

| Model | Hyperparameter | Search space |
|---|---|---|
| Linear Regression (LR) | Regularization strength ($C$) | {0.01, 0.1, 1, 10} |
| GPS | Learning rate | $\{10^{-4}, 10^{-3}, 10^{-2}\}$ |
| | L2 regularization | $\{10^{-5}, 10^{-4}, 10^{-3}\}$ |
| | Hidden layer size | {32, 64, 128, 256} |
| $E_{\text{max}}$ (dose–response) | Maximum effect ($E_{\max}$) | [0, 10] |
| | Half-max dose ($ED_{50}$) | Within observed dose range |
| | Hill coefficient | {0.5, 1, 2} |
| CausalPhysioNet | Hidden layer width | {64, 128, 256} |
| | Learning rate | $\{10^{-4}, 10^{-3}\}$ |
| | Batch size | {32, 64} |
| | Dropout rate | {0.2, 0.5} |
| | Independence regularization weight | {0.1, 0.25, 0.5} |

**Table S2.** Hyperparameter search spaces used for model selection. Hyperparameters were tuned using randomized or grid search with 5-fold cross-validation on a factual validation set.

## 4 ADDITIONAL ANALYSIS ON COVERAGE COUNTERFACTUAL PROXY SET

To contextualize the findings on the counterfactual set, we also assessed the degree to which the matched validation set provides coverage across the treatment range. Adequate coverage is necessary for meaningful counterfactual evaluation, as sparse representation at certain dose levels limits the reliability of performance estimates. As shown in Figure S3, the dosage distribution is highly right-skewed, with a concentration of observations at low physiotherapy intensities (0–10 minutes) and progressively fewer observations at higher doses. Nonetheless, non-zero coverage extends up to approximately 120 minutes. This pattern suggests that counterfactual performance estimates are most reliable at lower dosage ranges, where empirical support is greatest.

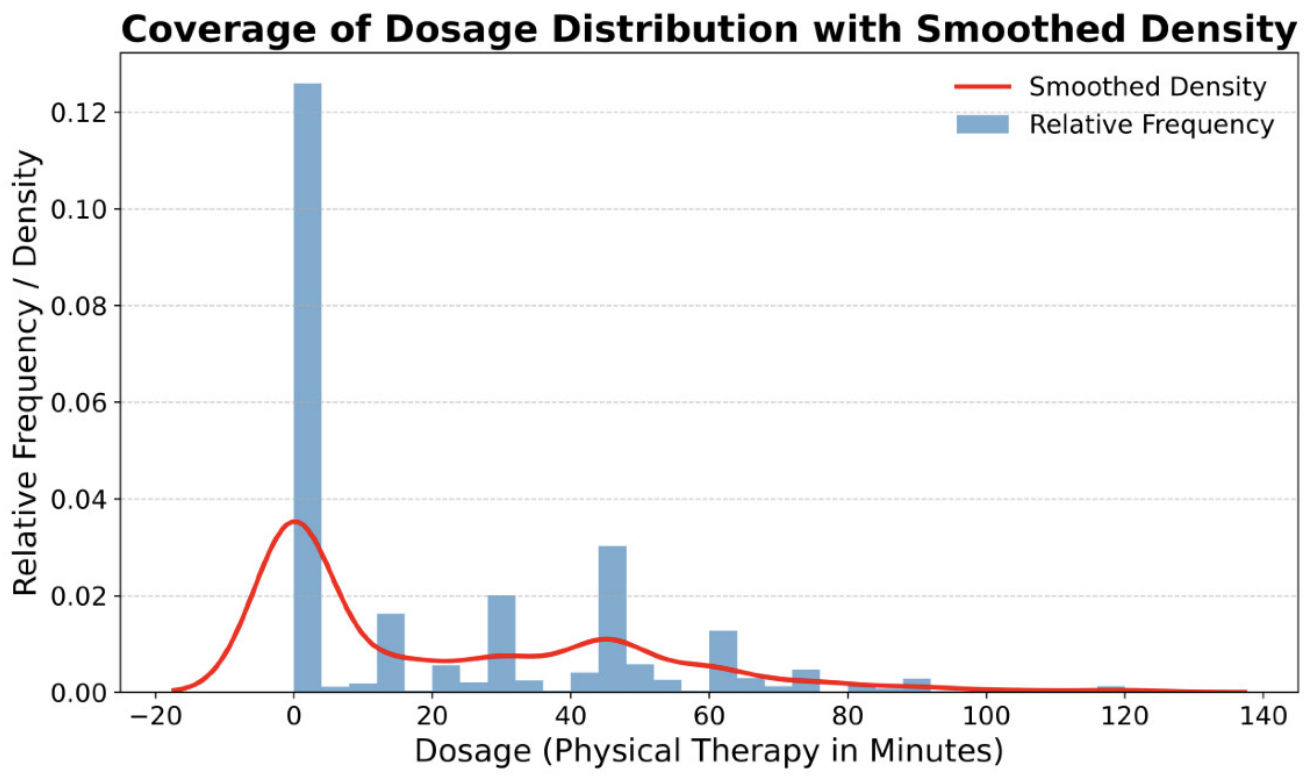


**Figure S3.** Coverage of the dosage distribution in the counterfactual validation set. The histogram (blue) depicts the relative frequency of physiotherapy dosages (in minutes) for the matched items, and the red curve shows the corresponding smoothed density estimate.
